# The application of Borg scale in cardiopulmonary resuscitation: An integrative review

**DOI:** 10.1371/journal.pdig.0000592

**Published:** 2024-08-28

**Authors:** Lucia Tobase, Sandra Helena Cardoso, Renata Tavares Franco Rodrigues, Dhieizom Rodrigo de Souza, Debora Gugelmin-Almeida, Thatiane Facholi Polastri, Heloisa Helena Ciqueto Peres, Sergio Timerman

**Affiliations:** 1 São Camilo University Center, São Paulo, Brazil; 2 School of Nursing at the University of São Paulo, Brazil; 3 Heart Institute of the Hospital das Clínicas of the Faculty of Medicine of University of São Paulo, São Paulo, Brazil; 4 Bournemouth University, Bournemouth, England; University of Reading Reading School of Pharmacy, UNITED KINGDOM OF GREAT BRITAIN AND NORTHERN IRELAND

## Abstract

The study of human performance and perception of exertion constitutes a fundamental aspect for monitoring health implications and enhancing training outcomes such as cardiopulmonary resuscitation (CPR). It involves gaining insights into the varied responses and tolerance levels exhibited by individuals engaging in physical activities. To measure perception of exertion, many tools are available, including the Borg scale. In order to evaluate how the Borg scale is being used during CPR attempts, this integrative review was carried out between October/2020 and December/2023, with searches from PubMed, CINAHL, Web of Science, Embase, PsycINFO and VHL. Full publications relevant to the PICO strategy were included and letters, editorials, abstracts, and unpublished studies were excluded. In total, 34 articles were selected and categorised into three themes: a) CPR performed in different contexts; b) CPR performed in different cycles, positions, and techniques; c) CPR performed with additional technological resources. Because CPR performance is considered a strenuous physical activity, the Borg scale was used in each study to evaluate perception of exertion. The results identified that the Borg scale has been used during CPR in different contexts. It is a quick, low-cost, and easy-to-apply tool that provides important indicators that may affect CPR quality, such as perception of exertion, likely improving performance and potentially increasing the chances of survival.

## Introduction

Understanding human performance and the way exertion is perceived, is crucial for monitoring health implications and improving training results. This includes gaining awareness of the diverse responses and tolerance levels displayed by individuals engaging in similar physical activities [1]. In the 1960s, Gunmar Borg developed the Rating of Perceived Exertion (RPE) scale [2] to quantify the subjective perception of effort, serving as a tool to measure the difficulty and strenuousness of performing physical activities. This perception is unique to each individual and is influenced by factors such as health status, age, physical and environmental conditions [3]. The scale is based on the idea that an individual’s perception of exertion during exercise is closely linked to their physiological responses such as increased heart and respiratory rate, sweating and muscle fatigue [4]. These responses serve as indicators of the body’s physiological adaptation to the demands of the exercise. The RPE scale (6–20) ranges from six to 20, where six indicates “no exertion” and 20 represents “maximal exertion”. Participants are asked to rate their perceived level of exertion during exercise, with the number on the scale that best represents their perception. This subjective assessment is important as it provides valuable information about an individual’s tolerance, comfort, and overall experience during physical activity. The RPE scale is a low-cost tool, easy to understand and apply, and validated in multiple contexts [3]. Following this, Borg introduced the CR scale (CR10), spanning from 0, 0.5, 1 to 10, serving to evaluate exertion, pain, and dyspnea [1]. Referred to as Borg CR-10, it is also recognised under various names such as Borg Dyspnea scale, Angina scale, Fatigue scale, Anxiety scale, and Pain scale [5,6]. The tool has also been validated in Brazil to assess vocal effort [7]. Subsequently, in 1982, the CentiMax scale (CR100) emerged, encompassing a range from 0 to 100 and functioning as a psychological measure for the assessment of depression [5,8,9].

The perceived exertion enables researchers to understand the impact of work intensity in areas such as emergency care. Interventions such as cardiopulmonary resuscitation (CPR) require immediate response, with high demands on performance and physical effort. Despite advances in the field of CPR, survival to hospital discharge rates is still low, making cardiac arrest a worldwide health challenge with high rates of morbidity, mortality, and associated costs [10]. According to the American Heart Association (AHA) guidelines, the chances of survival are directly associated with the quality of CPR [11]. It depends, among other factors, on the rescuer’s performance during chest compressions, which is considered a strenuous physical activity, but crucial to establishing coronary perfusion pressure, in an attempt to promote the return of spontaneous circulation [11].

In this context, to bridge the knowledge gap around perception of exertion in relation to CPR quality, this study aims to evaluate the effectiveness of using the Borg scale during CPR performance.

## Methods

Integrative review carried out from October 2020 to December 2023, with initial definition of the problem and research focus, comprehensive literature search, evaluation and critical analysis of data, and presentation of results [12]. Search of the published and unpublished literature was performed based on the guiding question “How is the Borg scale being used during cardiopulmonary resuscitation attempts?” Six electronic databases were used to identify eligible studies: PubMed, CINAHL, Web of Science, Embase, PsycINFO and the Virtual Health Library (VHL) portal, using an institutional Virtual Private Network.

Full publications of primary studies relevant to the PICO strategy were included and letters, editorials, abstracts, and unpublished studies were excluded. No time or language limit were established, to avoid compromising the sensitivity of the searches.

During descriptor selection, it was observed that there were no terms directly linked to the Borg scale. Notably, when controlled descriptors were combined without reference to the scale, a substantial decrease in search results was evident. However, incorporating "Borg scale" significantly facilitated the discovery of a more considerable number of relevant studies for this review.

To effectively structure the descriptors, the following PICO strategy (Population, Intervention, Context and Outcome) was used:

P = person of any age performing CPR;

I = Borg scale in the analysis of perceived exertion during simulated or real-life CPR;

C = simulated or real-life cardiorespiratory arrest.

O = perception of exertion

A pre-defined search strategy was used combining Boolean operators ‘AND’ and ‘OR’ with medical search headings and subheadings (e.g. MeSH) when applicable (S1 Appendix).

In order to facilitate the recording and analysis of eligible sources, each identified study from the initial search was collated within a group in EndNote web [13], with any duplicates removed. Subsequently, the search protocol was structured in an Excel spreadsheet to extract the following data: study title, author, year of publication, country, journal, database, objective, method, population, interventions, and results. The data search and extraction process were carried out by three independent reviewers (LT, SHC, RTFR) and conflicts in the analyses were mitigated by the other reviewers. Due to the nature of this review, risk of bias assessment was not performed [14].

## Results

**Fig 1:**
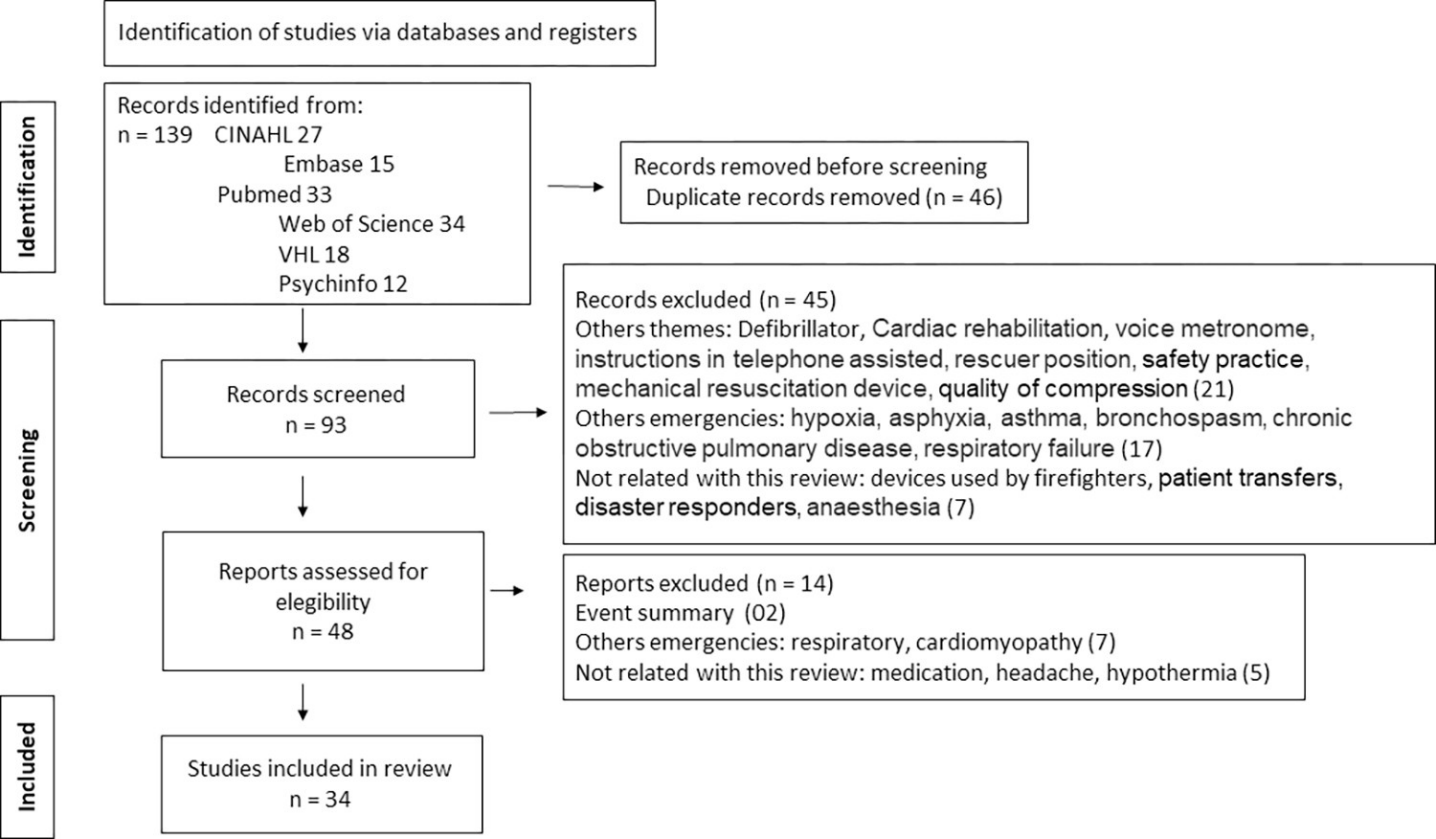
PRISMA—Preferred Reporting Items for Systematic Reviews and Meta-Analyses: the PRISMA statement.

Studies were presented according to the PRISMA (Preferred Reporting Items for Systematic Reviews and Meta-Analyses) diagram [15], as shown in Fig 1.

Of the 139 studies initially identified, 34 studies met the inclusion criteria and were included in this review after full text analysis. The number of included sources according to each database was: eight (23%) in VHL [16–23], six (18%) in CINAHL [24–29], seven (21%) in PubMed [30–36], four (12%) in Embase [37–40] and nine (26%) in Web of Science [41–49]. The year of publication ranged between 2008 and 2023. Country of publication included: Austria [18,20,21,30,32,33,35,41], United States [17,23,24,29,46], Canada [31,42,44], China [16,25,40,49], Japan [34,36], Spain [22,27,47], France [39], United Kingdom [19], Czech Republic [43], Taiwan [26,48], Hungary [28], Korea [37], Saudi Arabia [45] and Brazil [38].

Most studies were randomised crossover studies [16,18,20,24–27,29–31,36,37,39,41–47,49]. Other methods included randomised controlled trials [17,21,23,28,32,33,35,38], non-randomised controlled studies [22,34], and observational studies [19,40,48].

When evaluating perceived exertion with the Borg scale, 22 studies used RPE (6–20) [17,19–24,27,29–34,38,40–43,46,48,49], 11 used CR10 [16,18,25,26,28,35,36,37,39,44,47] and one used CR100 [45]. Due to the heterogeneity of the articles analysed, we classified the studies into three distinct groups: a) CPR performed in different contexts (e.g. simulation, high altitude, microgravity, helicopter) (Table 1); b) CPR performed in different cycles, positions, and techniques (Table 2); c) CPR performed with additional technological resources (e.g. mechanical CPR, metronome, telephone, video) (Table 3).

**Table 1:** Studies investigating the application of the Borg scale with CPR performed in different contexts.

Author Country, year	Study type	Sample size	Intervention	Outcome measures	Results
Niederer et al. [18] Austria, 2023	Randomized crossover	20 mountaineers	BLS simulation for 16 min at baseline altitude and at high altitude. Borg scale (CR10)	Vital signs, perception of fatigue.	Significant decrease in oxygen saturation from 97% (SD2%) at baseline to 87% (SD3%) at high altitude (p<0.01). After 16 min of CPR, heart rate had significantly increased [119 bpm (SD 12 bpm) to 124 bpm (95%CI −1.59 to 12.19). No significant difference in perception of fatigue between baseline and high-altitude (2.7 (SD 1.1) vs. 2.6 (SD 0.8), p>0.05).
Manoukian et al. [29] USA, 2022	Randomized crossover	3 ALS physicians	CPR in a moving vehicle (3x 2-min stable drive and 3x 2-min dynamic drive). Borg scale (6–20)	CPR variables and perceived fatigue.	Stable drive had significantly better CPR score for rate and recoil compared to dynamic drive. There was no significant difference for other measured CPR metrics. Perceived fatigue was greater for dynamic drive (8±1 vs 3.5±1.53) (p = 0.02).
Rehnberg et al. [19] United Kingdom, 2011	Observational	21 men	Evetts Russomano (ER) method during CPR in simulated microgravity. Borg scale (6–20)	Heart rate, perceived exertion rate and arm flexion angle.	Heart rate, perceived exertion, and elbow flexion of both arms increased using the ER method.
Barcala-Furelos et al. [22] Spain, 2016	Non-randomized controlled	23 lifeguards	Rescue and CPR on drowning victims, with *vs* without rescue equipment. Borg scale (6–20)	Rescue time, quality CPR and perceived exertion.	Shorter total rescue time with equipment (p <0.001). Increased chest compression rate with rescue tube (p <0.01). Less effort using a rescue board (p <0.001).
Asselin et al. [23] USA, 2018	Randomized controlled trial	40 clinicians	20 teams performed 3 simulations: (baseline; repeat in the same role; repeat in reversed roles). Experimental groups used RTF for simulations 2 and 3. Borg scale (6–20)	Heart rate, amylase, energy expenditure (NASA-TLX), perceived exertion and CPR quality.	No difference in % heart rate, salivary amylase and NASA-TLX between groups. Reduced levels of physical exertion and perceived workload than control subjects. Positive but limited impact on the quality of CPR performance.
Banfai et al. [28] Hungary, 2022	Randomized controlled trial	216 healthcare students	Continuous CPR for 2 minutes, using a surgical vs. fabric mask. Borg scale (CR10)	Vital parameters and perception of fatigue	No significant difference in changes in vital parameters and perception of fatigue.
Havel et al. [30] Austria, 2011	Randomized crossover	24 certified ALS	ALS in moving ambulance *vs* flying helicopter. Borg scale (6–20)	Blood pressure; serum lactate concentrations; Nine Hole Peg Test; perception of exertion	No significant difference in blood pressure, serum lactate and modified Nine Hole Peg Test. Significant reduction on the Borg scale by 0.89 points (95% CI = 0.42–1.350) (p <0.001) with feedback device.
Sato et al. [34] Japan, 2018	Non-randomized controlled	24 volunteers	Conventional CPR *vs* compression only in a hypoxemic environment, simulating high altitudes. Borg scale (6–20)	Quality CPR and perception of fatigue	Deterioration in performance for compression only CPR. Borg scale after 8 min CPR: greater perceived exertion and fatigue inside the hypobaric chamber and lower outside the chamber (15 ± 2 *vs* 11 ± 2; p <0.01 in paired t-test).
Egger et al. [35] Austria, 2020	Randomized controlled trial	20 climbers	CPR (30:2) in high altitude Borg scale (CR10)	Quality CPR, heart rate, SpO2 and perceived exertion	Decrease in the mean depth of chest compression (95% CI 0.5 to 1.3; p <0.01), with no difference in compression rate. Increase in heart rate, reduction in SpO_2_. Increased perception of fatigue after 2 minutes of CPR.
Nakashima et al. [36] Japan, 2020	Randomized crossover	45 professionals and medical students	CPR whilst walking alongside a conventional stretcher vs walking alongside a stretcher with support (“wings” method) Borg scale (CR10).	Quality of the chest compressions and perception of fatigue	Higher average compression rate and depth (p <0.01), with better quality and less perception of fatigue using stretchers with wings.
Ahn et al. [37] Korea, 2021	Randomized crossover	30 BLS and ALS providers	4-min continuous compressions on a mannequin: on a flat floor and 3 types of mattresses (soft, medium, hard). Borg scale (CR10)	Vital parameters and perception of fatigue	No significant differences in vital parameters. Perceived exertion was lower when compressions were performed on the floor (p = 0.003)
Havel et al. [41] Austria, 2008	Randomized crossover	24 ALS providers	CPR for 8 min in moving ambulance *vs* flying helicopter. Borg scale (6–20).	Heart rate to blood pressure ratio; blood pressure, serum lactate, Nine Hole Peg Test, and perceived exertion.	Mean heart rate to blood pressure ratio was smaller (0.89 ± 0.21) in the ambulance compared to (1.01 ± 0.21) in the flying helicopter (p = 0.04). No significant difference in other physiological parameters. Perceived exertion increased in all groups.
Pompa et al. [44] Canada, 2019	Randomized crossover	18 clinicians	Conventional CPR *vs*. Koch compression, in a helicopter. Borg scale (CR10).	CPR quality and perceived exertion.	CPR overall quality was 63% in conventional compressions *vs* 79% with Koch compressions (p = 0.04). Significant reductions in physical exertion for Koch compressions (p < 0.001).
Kingston et al. [45] Saudi Arabia, 2021	Randomized crossover	27 ALS providers	Chest compressions for 10 min on a mannequin on concrete and on foam. Borg scale (CR100)	Heart rate, quality of compressions and perception of fatigue	No effect on heart rate (p = 0.143). Significant difference (p = 0.019) in compression depth (≥50mm). Perceived fatigue was lower (p <0.001) when CPR was performed on concrete floor.

**Table 2:** Studies investigating the use of the Borg scale when CPR is performed in different cycles, positions, and techniques.

Author Country, year	Study type	Sample size	Intervention	Outcome measures	Results
Chi et al. [16] China, 2008	Randomized crossover	18 healthcare professionals	5-min CPR in 3 positions: kneeling, standing next to the manikin on the table and standing next to the manikin on a lower table. Borg scale (CR10).	Quality CPR and perception of fatigue	No significant difference in quality of CPR (p> 0.05). No significant difference in perceived fatigue between the three positions.
Trowbridge et al. [24] USA, 2009	Randomized crossover	20 female lay volunteers	Continuous chest compression vs 30:2 cycle. Borg scale (6–20).	Compression depth, rate, metabolic fatigue, and perceived exertion at 5 minutes and at the end of CPR	Average depth and rate were significantly lower (p < .004) and (p < 0.001) for continuous CPR. Metabolic fatigue was significantly greater for continuous CPR (p = 0.02). Across both groups, perception of fatigue increased at the end of CPR (p < .001).
Chi et al. [25] China, 2010	Randomized crossover	17 professionals	CPR in different cycles: 15:2; 30:2 and 50:5, for 5 min with 50-min rest. Borg scale (CR10)	Perception of fatigue and discomfort in the body area.	Perception of fatigue was significantly higher (p = .008) in the 50:5 (3.67±2.04) when compared to 15:2 (2.20±1.740) and 30:2 (2.76±1.73). Waist discomfort was noted and was significantly higher in 50:5 (1.83±1.79) when compared to 15:2 (1.40±1.63) and 30:2 (1.56±1.28) (p = .024).
Tsou et al. [26] Taiwan, 2022	Randomized crossover	35 nurses	Paediatric chest compressions with one and two hands for 2 minutes. Borg scale (CR10)	Perception of exertion and pain.	Perceived exertion and pain for one-handed compressions were significantly higher than with two-hands (p< 0.001 and p = 0.004 respectively).
Barcala-Furelos [27] Spain, 2022	Randomized crossover	58 lifeguards	Simulated infant CPR in pairs for 20 minutes using two-fingers and two-thumbs technique. Borg scale (6–20)	Perception of fatigue	Perception of fatigue is higher in the two-finger technique compared to the two-thumb technique (p = 0.01).
Vaillancourt et al. [31] Canada, 2011	Randomized crossover	42 participants ≥55 years old	CPR (30:2 and 15:2) for 5 min and rest for 5 min. Borg scale (6–20)	Heart rate, blood pressure, venous lactate, and perceived exertion	No significant difference in physiologic measures. Higher level of fatigue using a 30:2 compared to a 15:2 but not statistically significant.
Van Tulder et al. [32] Austria, 2014	Randomized controlled trial	26 adults	CPR with dispatcher recommending "compress firmly" *vs*. "compress as hard as you can", for 10 min. Borg scale (6–20)	Compression depth and perception of fatigue	Mean compression depth and perception of fatigue were not significantly different between groups (p = 0.66) and (p = 0.89).
Skulec et al. [43] Czech Republic, 2016	Randomized crossover	Ten volunteers	Continuous CPR *vs* 30:2 for 30 min. Borg scale (6–20)	Oxygen consumption and perception of exertion and perception of fatigue	Greater oxygen consumption (p = 0.049) for compressions-only CPR. Higher perceived exertion (p = 0.001) and perceived fatigue (p = 0.058) with compression-only CPR.
Marquis et al. [39] France, 2023	Randomized crossover	100 EMS participants	Chest compressions with overlapping or interlocking hands. Borg scale (CR10)	Overall chest compressions success score, CPR metrics and perception of exertion	Median chest compression score: 79.5% IQR[48.5–94.0] in the overlapping hands group and 71% IQR[38.0–92.8] in the interlocking hands group (p = 0.37). No significant difference for CPR metrics or perception of exertion.
Santos-Folgar et al. [47] Spain, 2022	Randomized crossover	21 university students	2-min standard pediatric CPR vs walking with a dummy on the forearm. Borg scale (CR10)	CPR quality and perceived exertion	Standard pediatric CPR showed higher overall quality (59% vs 49%; P = 0.02) Ambulating paediatric CPR had a higher perceived exertion (2 vs 5; P< 0.001).
Chang et al. [48] Taiwan, 2021	Observational	70 firefighters	3 CPR tests: (i) uninterrupted for 10 minutes; (ii) after 2 days rest, 5 cycles of 2-min CPR with 10s rest; (iii) after 2 days rest, 5 cycles of 2-min CPR with 20s rest Borg scale (6–20)	CPR performance and perceived exertion	No significant differences in compression depth or rate among the three methods (p > 0.05). Perceived exertion during uninterrupted CPR was significantly higher (p < 0.001).
Dong et al. [49] China, 2021	Randomized crossover	28 lay people	Hands-only CPR with different rest intervals. Borg scale (6–20).	Average chest compression depth, vital signs, and perceived fatigue	Significant impact on chest compression depth between different intervals (p = 0.045). No significant difference among all methods in any physiological indicators or in perception of fatigue.

**Table 3:** Studies investigating the use of the Borg scale when CPR is performed with additional technological resources.

Author Country, year	Study type	Sample size	Intervention	Outcome measures	Results
Barash et al. [17] USA, 2011	Randomized controlled trial	30 BLS/ALS participants	15 pairs performing simulated CPR, with a change of role (chest compression and AED management), every 2 CPR cycles. With and without automated feedback device. Borg scale (6–20).	Pre-shock pause time and perceived exertion.	Pre-shock pause time was reduced by 80%, with automated feedback (p<0.0001). No difference in perceived exertion.
Fischer et al. [20] Austria, 2012	Randomized crossover	80 medical students	CPR for 12 min with manual mechanical resuscitation device (MRD) *vs* standard BLS. Borg scale (6–20)	Heart rate, capillary lactate and perception of exertion	Heart rate increased in the final minute of standard BLS (p = 0.027). Mean serum lactate concentration decreased with MRD (p≤0.001). Perception of fatigue increased with MRD *(*p = 0.027).
Van Tulder et al. [21] Austria, 2014	Randomized controlled trial	32 volunteers	CPR for 10 min with instructions over the phone with standard wording ("push down firmly 5 cm"), or intensified wording ("it is very important to push down 5 cm every time") Borg scale (6–20).	Chest compression depth, vital signs reflecting physical strain, perception of fatigue	Compression depth, vital signs and perception of fatigue were not significantly different between groups.
Van Tulder et al. [33] Austria, 2015	Randomized controlled trial	36 volunteers	Simulated CPR for 10 min with conventional metronome or continuous voice metronome. Borg scale (6–20).	CPR quality, heart rate, blood pressure, nine-hole peg test, and perception of fatigue	No significant difference in CPR quality, physiological parameters, and perception of fatigue.
Tobase et al. [38] Brazil, 2023	Randomized controlled trial	69 nurses	Simulated BLS with or without feedback device. Borg scale (6–20)	Heart rate and perceived exertion	Heart rate and perceived exertion were significant lower with feedback device (p<0.001).
Li et al. [40] China, 2023	Observational	100 lay adults	3x 2-min cycles of simulated continuous CPR: without video and with video after 72h rest. Borg scale (6–20)	CPR metrics and perception of fatigue	Every CPR metric improved significantly with video (all p<0.001). Perception of fatigue significantly increased with video (p< 0.001).
Liu et al. [42] Canada, 2016	Randomized crossover	63 participants ≥ 55 years old	30:2 and continuous CPR for 5 minutes, with a metronome. Borg scale (6–20).	CPR quality and perception of fatigue	More adequate chest compressions (p = 0.0001) with continuous CPR. No significant difference in perception of fatigue.
Manoukian et al. [46] USA, 2022	Randomized crossover	15 firefighters	Manual and mechanical CPR for 2 minutes, on a boat with stable (linear) and dynamic (curved) navigation. Borg scale (6–20)	Quality of compressions and perception of fatigue	Mechanical CPR favoured the quality of compressions and perception of fatigue (<0.001).

### CPR performed in different contexts

Under this category, 14 studies applied the Borg scale during CPR being performed in different contexts such high altitude [18,34,35], microgravity [19], in the water [22], in helicopter or moving vehicles [29,30,41,44], on different surfaces [36,37,45], wearing face mask [28], and following different protocols [23], as demonstrated in Table 1.

### CPR performed in different cycles, positions and techniques

Six studies [24,25,31,43,48,49] used the Borg scale during CPR in different cycles including 15: 2, 30:2, 50:5 and continuous chest compression. Two studies [16,47] applied the Borg scale when investigating CPR being performed in different positions such as kneeling, standing, bending on a low surface, or walking; and four studies applied the tool during different CPR techniques [26,27,32,39], as demonstrated in Table 2.

### CPR performed with additional technological resources

In this category, eight studies applied the Borg scale when CPR was provided in combination with mechanical CPR [20,46], phone-assisted CPR [21], with the use of AED [17], teaching video [40] and using feedback devices [33,38,42], as demonstrated in Table 3.

## Discussion

The aim of this study was to evaluate the effectiveness of using the Borg scale during CPR performance in both simulation and real-life contexts. Using the Borg scale during CPR can aid in monitoring and managing the rescuer’s exertion levels, which is crucial for preventing exhaustion and sustaining effective compressions, thus reducing the risk of injuries, and ensuring consistent CPR quality.

It was observed in this review that the Borg scale has been used in several contexts related to CPR to measure the perception of fatigue and/or exertion, with effective contributions to analysis of physical demands. This tool has been applied in other fields [50,51] and demonstrates that, due to the linear relationship between the Borg scale and physiological measures such as oxygen consumption, blood lactate concentration and heart rate during aerobic exercise or strength training, the scale can be a useful tool to monitor exercise intensity, either stationary or dynamic [1,6]. In the field of resuscitation, despite the heterogeneity of the articles included in this review, it has been evidenced that the application of the Borg scale is also beneficial to measure and understand physical demands related to CPR.

It was observed in this review that the Borg scale was applied during cardiac arrest in different scenarios and contexts, including simulation and pre-hospital care, where assistance in challenging environments influences rescuer’s performance during resuscitation [20]. Given the difficulty in carrying out research in real circumstances, a hypobaric chamber was used by Sato et al. (2018) to reproduce the hostile environment at high altitudes. Environments with a lower oxygen concentration require greater effort from the rescuer, negatively influencing the quality of CPR and the chances of survival [34,35]. Additionally, the greater physical and mental stress may result in feelings of fatigue and tiredness. The authors applied the Borg scale to rescuers providing CPR in the hypobaric chamber and scores obtained were higher than those at sea level due to the reduction in SpO_2_ and rise in heart rate, increasing the perceived exertion. Therefore, to reduce perceived exertion, the authors recommended changing rescuers every two minutes, particularly during CPR with continuous compression, aligning with the current resuscitation guidelines [11]. This is similar to the results from Niederer et al. [18] where mountaineers performed CPR in high altitude. Despite not finding a significant increase in perceived fatigue, physiological parameters such as oxygen saturation and heart rate increased significantly when CPR was performed 3454 meters above sea level. Based on the results, the authors suggest that it is possible to alternate rescuers every one minute in high altitude, where the hypoxic environment and the difficulty in providing CPR can lead to poor performance and physiological fatigue [18,34,35].

Barcala-Furelos and colleagues (2016) explored CPR in the water, evaluating the perception of exertion during compressions on drowning victims. The authors evaluated various rescue equipment to determine the safest option with the shortest rescue time and assessed the impact of these tools on lifeguards’ physiological conditions, perception of exertion and CPR performance. The authors suggest that the use of equipment reduced rescue time, particularly when using a rescue board. Additionally, perception of fatigue was significantly lower with the rescue board when compared to the other tools or without any equipment. However, the authors emphasise that, despite the benefits of using equipment for an improved rescue, there is a need for further training of lifeguards in the use of rescue boards and other tools [22].

In another simulation study exploring cardiac arrest in drowned children [27], lifeguards’ perception of exertion was greater when using the two-finger technique, when compared to the two-thumbs technique. Furthermore, when performing CPR with one-hand and two-hands in older children, the perception of exertion was lower when using both hands, instead of just one [26]. These results are complemented by the study performed by Santos-Folgar (2022) in the infant population. The authors used the Borg scale to evaluate perception of exertion when CPR was provided on an infant supported on the rescuer’s arm. Although it was observed that the quality of compressions was inferior when compared to standard CPR (i.e. infant placed on a hard surface), and the perception of exertion was higher, it is important to consider the need for rapid transport to the emergency department [47].

Performing CPR inside moving vehicles or aircrafts, such as ambulances and helicopters while transporting patients, can be a more complex intervention, which may impact the quality of resuscitation attempts and perception of fatigue [29,30,41,44]. Additional challenges are related to limited space, mobility constraints, vibrations and turbulences, safety concerns, communication difficulties, and equipment stability [52].

Adapting CPR techniques to these unique conditions is crucial, with a primary focus on ensuring the safety of both the patient and the healthcare provider. Interestingly, despite the above-mentioned challenges, a study by Havel et al. (2008) comparing physical effort during CPR performance in an ambulance and helicopter, did not show significant changes in physiological responses. Using the Borg scale (6–20), the authors concluded that the type of transport did not influence physical exertion, however, perception of fatigue increased throughout CPR performance [41], reinforcing the concept of changing rescuers every two minutes for improved performance. In a similar study, Pompa et al. (2019) suggested that changing the way chest compressions are provided is a possible alternative to mitigate the constraints of limited space and mobility [44]. This is also applicable when providing CPR in different positions is required (e.g. performing CPR kneeling, with a manikin on a table, or with overlapping hands) as alternative positions do not influence the perception of exertion, applied force and depth of compression during CPR [16,39]. Furthermore, if it is not possible to perform chest compressions with the hands, using the foot on the sternum can be an effective option, especially when the rescuer has no strength due to exhaustion, or is much smaller than the victim [42]. This is also applicable using the Evetts-Russoman method, where the rescuer’s legs are wrapped around the victim during CPR. Apart from providing adequate compression depth and rate [19], there seems to be a reduced perceived fatigue, potentially improving quality of CPR performance.

Ahn et al. (2021) applied the Borg scale to analyse the influence in quality when CPR is performed on a mattress. Although the authors have not found a significant difference in the quality of chest compressions when compared to CPR delivered on a hard surface, a greater perception of exertion and fatigue was observed [37]. This may be explained by the damping effect of mattress compressibility [53] where the surface may compress under the pressure of chest compressions, making it more difficult to achieve the recommended compression depth. Additionally, the softer surface of a mattress absorbs some of the energy generated during chest compressions, leading to energy dissipation, and reducing the force transmitted to the individual’s chest [54]. Despite not finding a significant difference in CPR performance, the increased perception of exertion can compromise the overall quality and duration of CPR, potentially impacting the patient’s chances of survival.

When comparing continuous compression cycles and standard CPR (e.g. 30:2), it was found that continuous cycles required greater effort, increasing fatigue levels [24]. Similarly, between 15:2 and 30:2 cycles, inadequate chest compressions and greater perception of fatigue were noticed in the latter [27], suggesting that the longer the cycle, the greater energy levels are needed [43], increasing perception of exertion. Chi and colleagues (2010) applied the Borg scale in cycles of 15:2, 30:2 and 50:5, and also concluded that CPR required moderate to heavy exertion progressively, after five minutes of activity [25]. The results of the abovementioned studies support the recommendation that switching rescuers every two minutes or less, improves maintenance of high-quality CPR performance [11]. This is particularly important when CPR is provided by an elderly person or with a slender build, as the constitution of the individual’s physical structure influences CPR performance, recovery time and perception of exertion [24,25,35,48,49]. Additionally, considering the correlation between perceived fatigue and increased heart rate, the application of the Borg scale can also be useful in monitoring CPR performance of individuals who take medications that affect heart rate [4], during prolonged duration of resuscitation attempts [41], for recovery and rehabilitation post-myocardial infarction [55], exertion in patients with pneumopathies [56,57], or long COVID-19 syndrome [58].

Current resuscitation guidelines recommend the use of feedback devices during CPR [11]. The tools provide verbal and/or visual information in real time about the quality and/or metrics of CPR [10] and are believed to reduce perception of exertion during resuscitation attempts [9,17,26,48]. Sound devices such as metronomes are useful in controlling the rhythm and frequency of chest compression [59], while audiovisual devices enable the rescuers to monitor the rhythm, depth, and release of compressions [60,61]. Applying the Borg scale with feedback devices provided additional insights into the rescuer’s perceived exertion levels, helping to ensure that the individual performing CPR can maintain a sustainable level of effort [38]. By combining the data from the feedback device with the subjective assessment provided by the Borg scale, the rescuer can make informed decisions about adjusting their CPR technique or intensity to optimise performance and maintain effective chest compressions. This integrated approach allows for a comprehensive evaluation of both objective and subjective parameters, contributing to enhanced CPR quality and potentially improving patient outcomes.

In addition to feedback devices, other technological resources such as mechanical resuscitation devices, remote guidance over the phone, or video-instruction have also been utilised during resuscitation attempts [20,21,32,33,40]. Although CPR performance may improve with the use of technological resources, when the Borg scale was applied in these circumstances, there were inconsistent conclusions regarding perception of exertion, with one study finding a significant difference when video-CPR was used [40], and others not finding statistically significant results [20,21,32,33]. Moreover, it was observed that quality of CPR performance may be negatively impacted, particularly when feedback devices are used in conjunction with telephone-assisted CPR [33]. Therefore, despite resuscitation guidelines encouraging the use of telephone guidance during CPR, especially for lay people [11], it is important to consider the individual’s profile and ability to understand the guidance and avoid the concomitant use of feedback device.

This review has highlighted the benefit of using the Borg scale to assess the level of exertion during CPR. Although it has been previously evidenced that there are different tools to analyse perception of exertion during physical activities, each offering unique approaches and insights (e.g. Visual Analogue scale, Likert scales) [1], the Borg scale is relatively easy to understand and apply, making it accessible for individuals of different educational backgrounds and age groups. For its straightforward nature that facilitates quick and accurate self-assessment of exertion levels during physical activities, the Borg scale has been recommended by several institutions such as the AHA [11], American College of Sports Medicine [62], and British Association for Cardiac Prevention and Rehabilitation [63].

It is important to recognise that, prior to application of the Borg scale, it is recommended that the tool and instructions on its criteria are presented in advance, so that users (adults or children) can familiarise themselves with its use and correct application [1,64].

### Limitations

This study has some limitations. First, the results of the included articles were obtained in a simulated environment, using a mannequin. CPR in a real situation can have other effects on the quality of performance and perception of exertion, possibly influenced by a higher level of stress. Second, potential biases were not systematically addressed like in a systematic review. Third, the heterogeneity among study design, population, outcome measures and Borg scale selected, may impact the interpretation and synthesis of the results. Fourth, it is important that the Borg scale be presented to the participants beforehand, in order to understand the respective scoring criteria and values, so that the response is as accurate as possible. However, not all studies described this particularity. Finally, the Hawthorne effect could have impacted the accuracy of results.

## Conclusion

The Borg scale was applied in different CPR contexts to analyse the rescuer’s perception of exertion during CPR performance. Identifying the factors that influence quality of performance such as perception of exertion and fatigue, can potentially contribute to enhancing CPR quality, inform resuscitation guidelines, and ultimately improve patient outcomes.

## Supporting information

S1 Checklist PRISMA Checklist. (DOCX)

S1 Appendix Search Strategy. (DOCX)
