# Supplementary material for: The application of Borg scale in cardiopulmonary resuscitation: An integrative review
Source: PLOS Digit Health. 2024 Aug 28;3(8):e0000592. doi: 10.1371/journal.pdig.0000592 (PMC11355535; doi:10.1371/journal.pdig.0000592)
Supplement: S1 Appendix — (DOCX) [file pdig.0000592.s002.docx]

**The application of Borg scale in cardiopulmonary resuscitation: an integrative review**

S1 Appendix

SEARCH STRATEGY:

CINAHL: (" *Borg scale* " OR " *perceived exertion* ") AND (" *cardiac arrest* " OR " *cardiopulmonary resuscitation* " OR *cpr* OR *resuscitation* );

EMBASE: ( *'Borg scale'* AND *'heart arrest'* /exp); PUBMED: ((" *Borg scale* " OR " *perceived exertion* ") AND (" *heart arrest* " OR " *cardiac arrest* " OR " *cardiopulmonary resuscitation*"));

Web of Science: ALL= ((" *Borg scale* " OR " *perceived exertion* ") AND (" *cardiac arrest* "));

PsycINFO: (" *Borg scale* " OR " *perceived exertion* ") AND *emergency* . Searches with “ *cardiac* *arrest* ” were ineffective, better results were found with *emergency* .

VHL: (“ *Borg scale* ” OR “ *perceived* *exertion* ”) AND “ *cardiac* *arrest* ”.
